# Supplementary figures and images for: Long-term patterns of an interconnected core marine microbiota
Source: Environ Microbiome. 2022 May 7;17:22. doi: 10.1186/s40793-022-00417-1 (PMC9080219; doi:10.1186/s40793-022-00417-1)

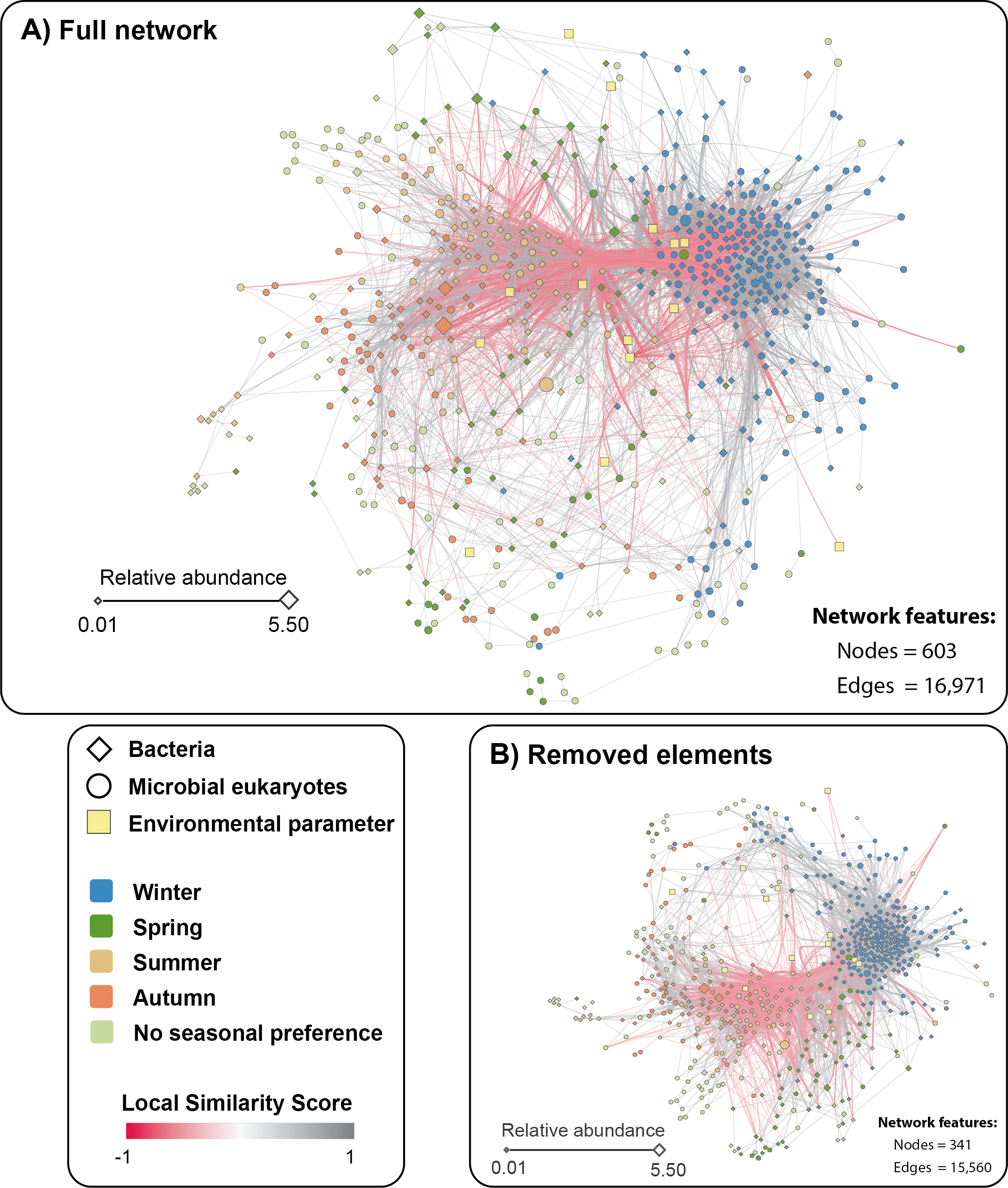

Supplement: Supplementary file 2 — Additional file 2: Figure S1. Panel A shows the full network constructed with the resident microbiota (that is, OTUs present in > 30% of the samples over 10 years; Table 1). Panel B displays network elements that were removed as they did not fulfill the cut-offs (that is, highly significant correlations (P & Q < 0.001), local similarity scores > |0.7| and Spearman correlations > |0.7|). [file 40793_2022_417_MOESM2_ESM.png]

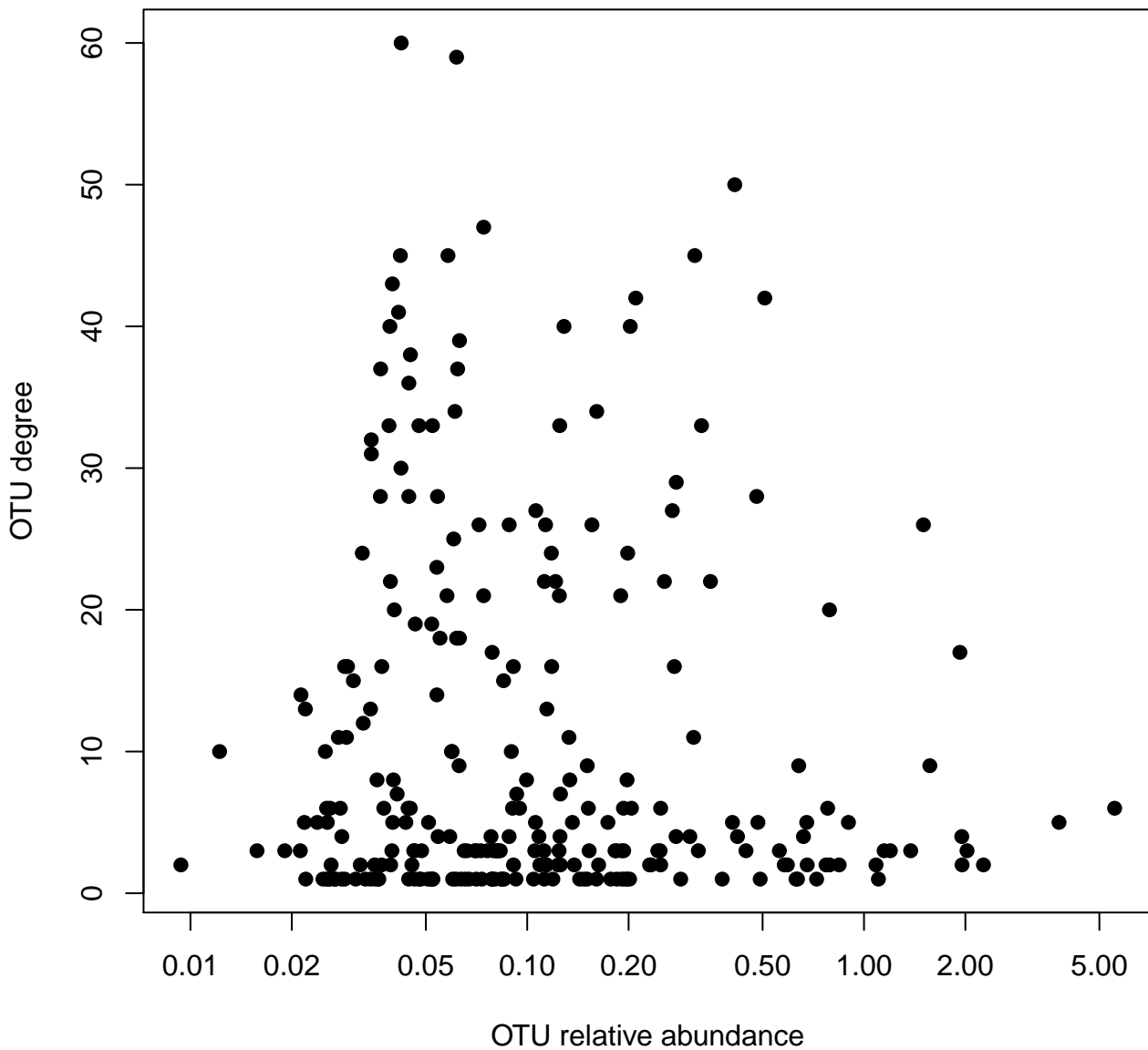

Supplement: Supplementary file 3 — Additional file 3: Figure S2. OTU relative abundance vs. degree shows no relationship in the core network. [file 40793_2022_417_MOESM3_ESM.pdf]

**Winter**

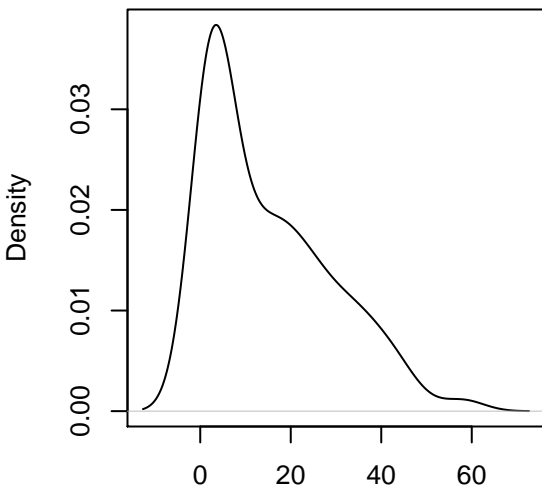

**Spring**

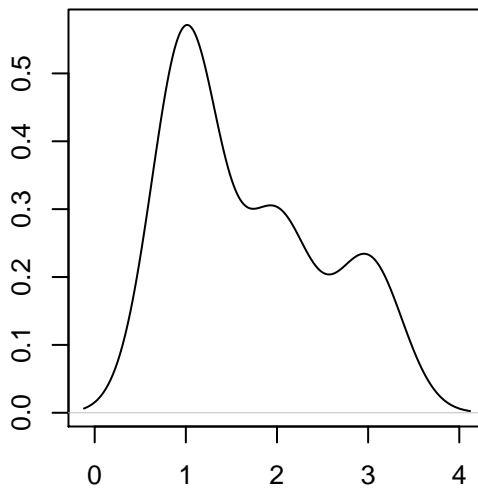

**Summer**

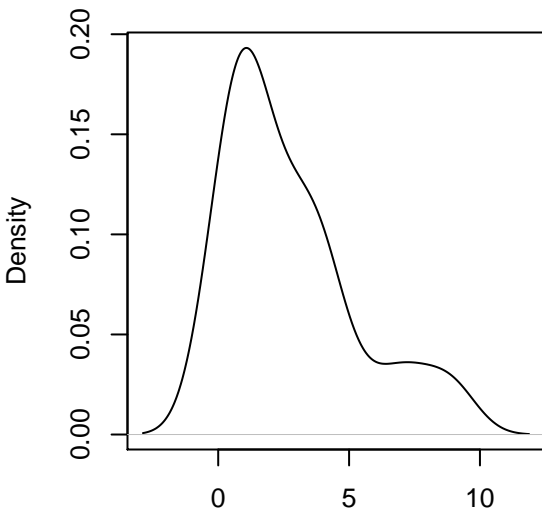

**Autumn**

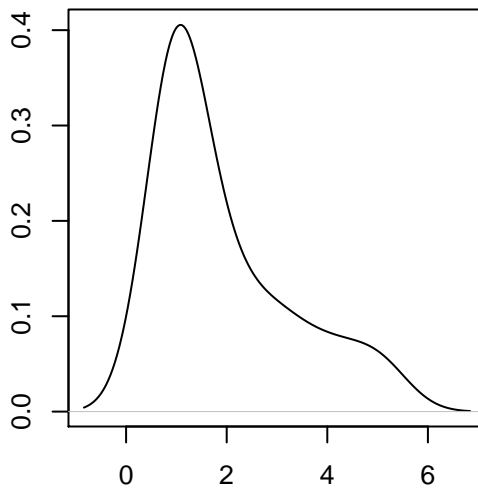

Supplement: Supplementary file 4 — Additional file 4: Figure S3. Distribution of the Degree values in the core network. [file 40793_2022_417_MOESM4_ESM.pdf]

**Winter**

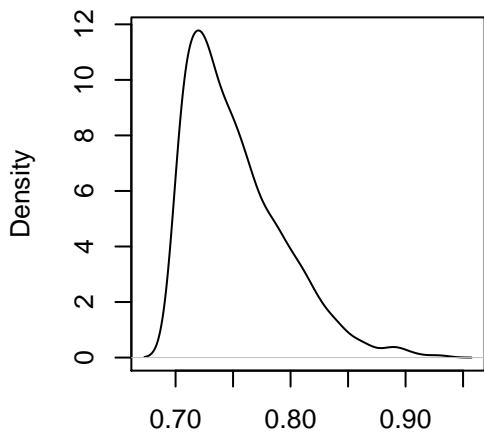

**Spring**

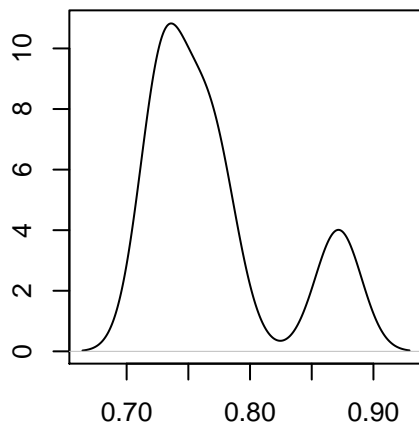

**Summer**

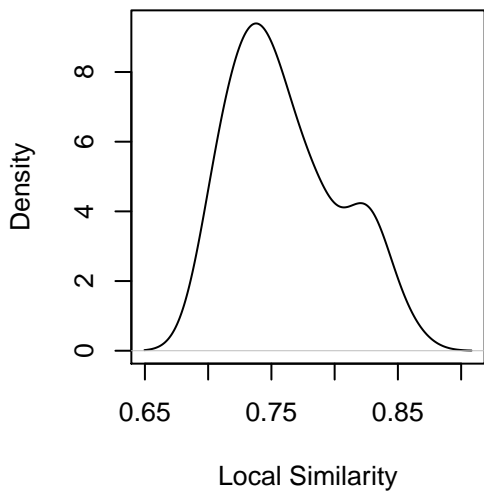

**Autumn**

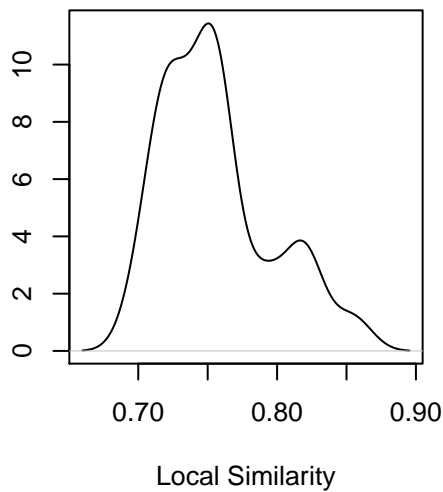

Supplement: Supplementary file 5 — Additional file 5: Figure S4. Distribution of the Local Similarity values in the core network. [file 40793_2022_417_MOESM5_ESM.pdf]

**Winter**

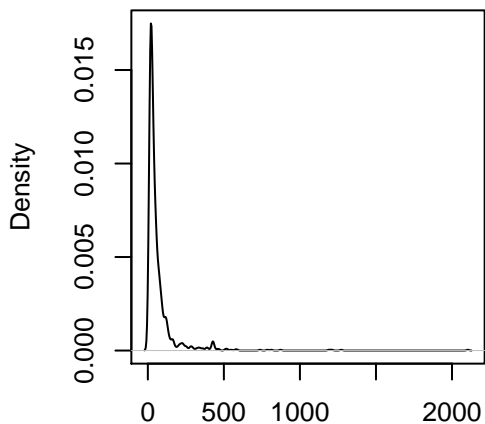

**Spring**

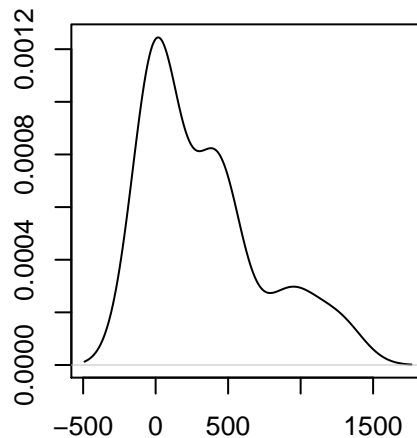

**Summer**

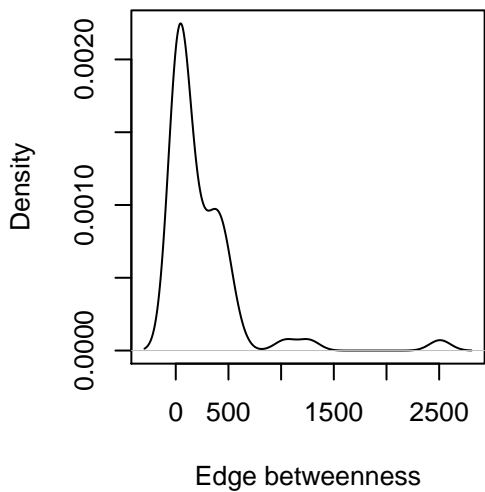

**Autumn**

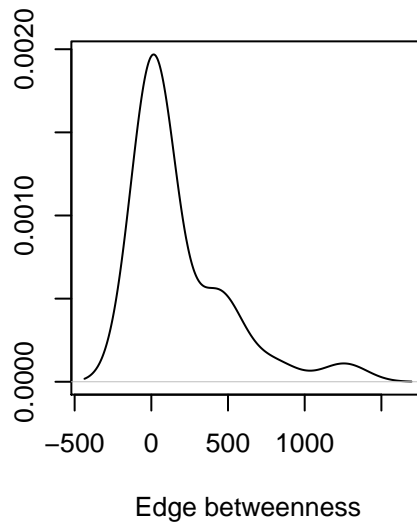

Supplement: Supplementary file 6 — Additional file 6: Figure S5. Distribution of the Edge betweenness values in the core network. [file 40793_2022_417_MOESM6_ESM.pdf]

**Winter**

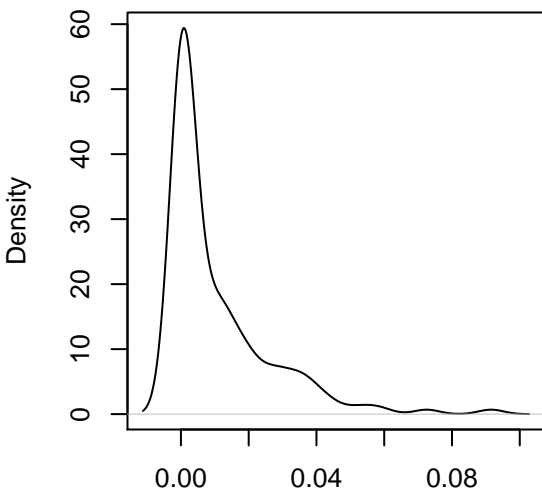

**Spring**

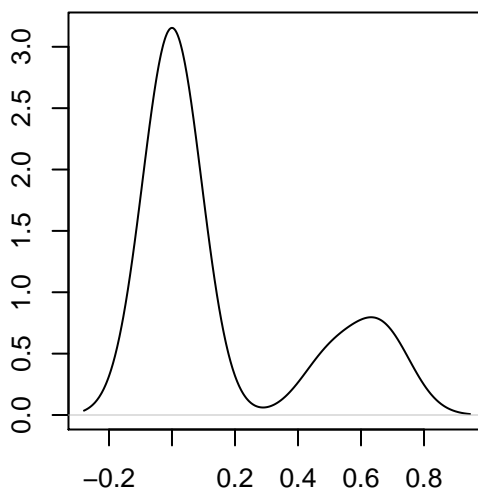

**Summer**

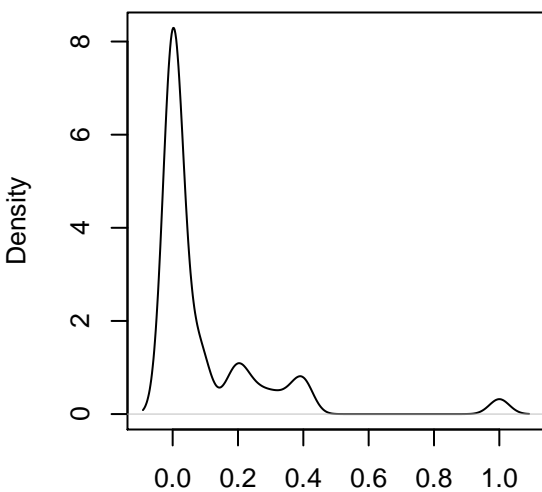

**Autumn**

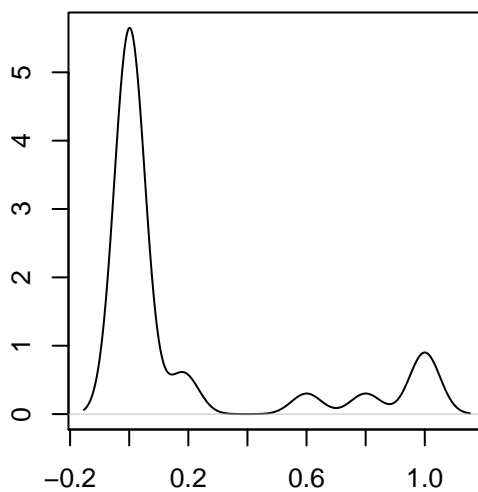

Betweenness centrality

Betweenness centrality

Supplement: Supplementary file 8 — Additional file 8: Figure S7. Distribution of the Betweenness centrality values in the core network. [file 40793_2022_417_MOESM8_ESM.pdf]

**Winter**

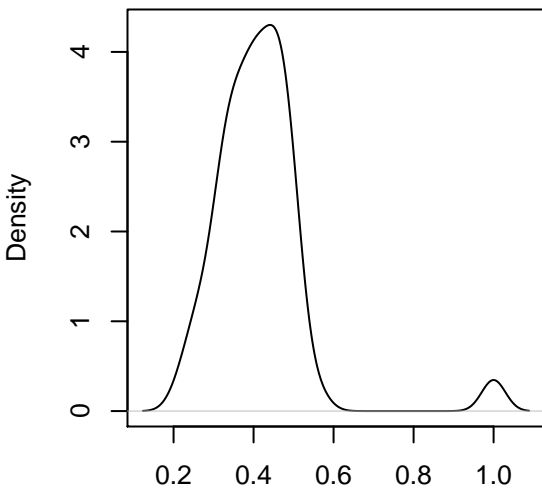

**Spring**

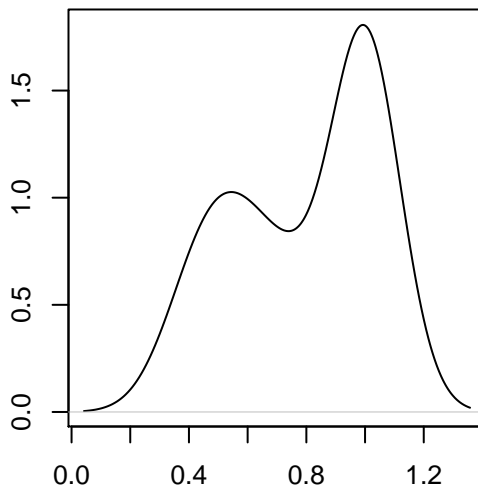

**Summer**

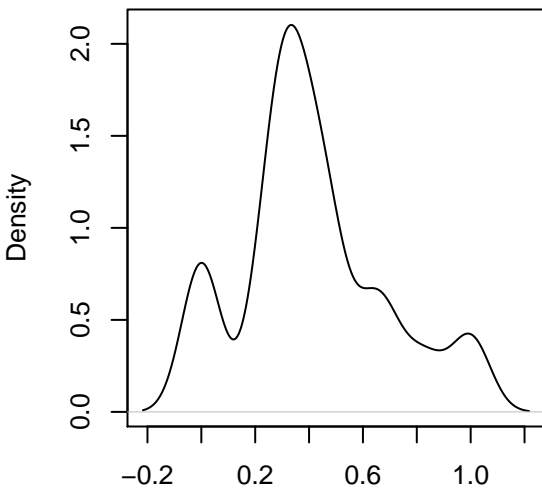

**Autumn**

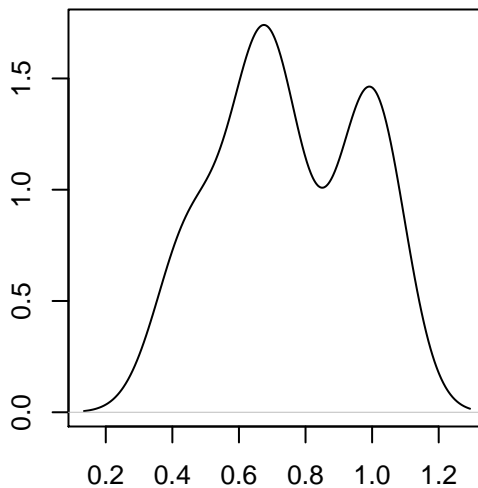

Supplement: Supplementary file 9 — Additional file 9: Figure S8. Distribution of the Closeness centrality values in the core network. [file 40793_2022_417_MOESM9_ESM.pdf]

**Winter**

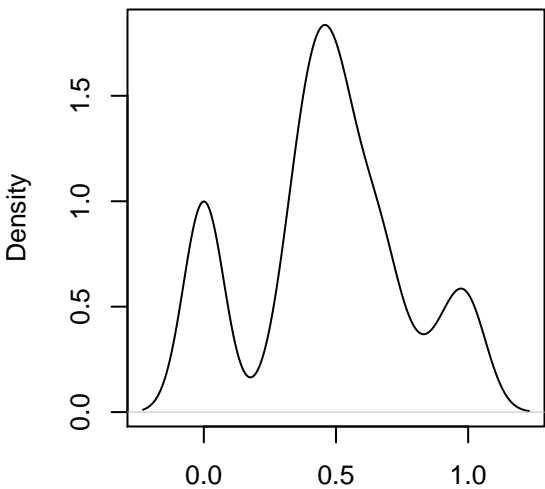

**Spring**

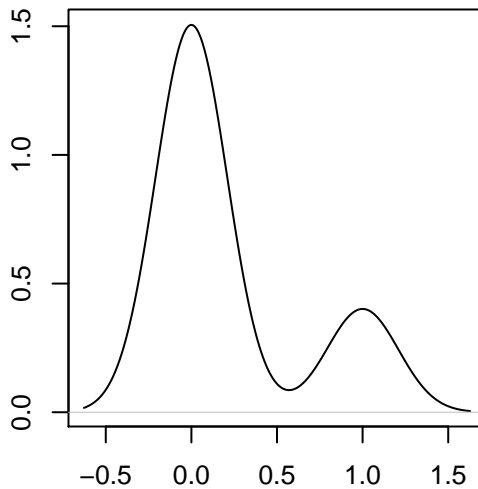

**Summer**

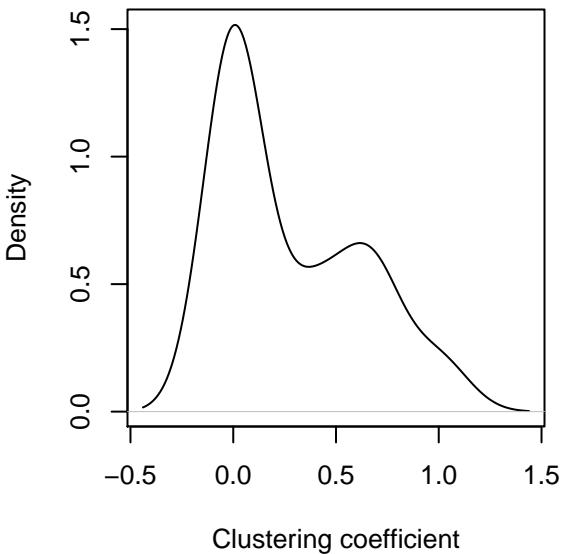

**Autumn**

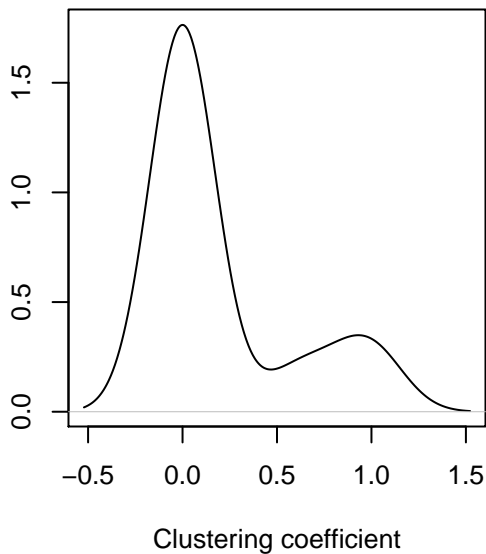

Supplement: Supplementary file 10 — Additional file 10: Figure S9. Distribution of the Clustering coefficient values in the core network. [file 40793_2022_417_MOESM10_ESM.pdf]

Resident Microbiota (709 OTUs)

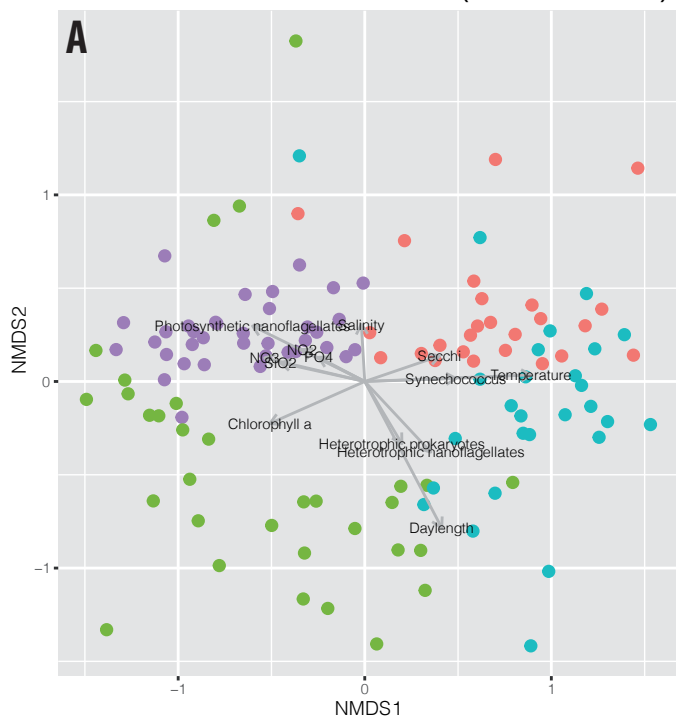

Core Microbiota (259 OTUs)

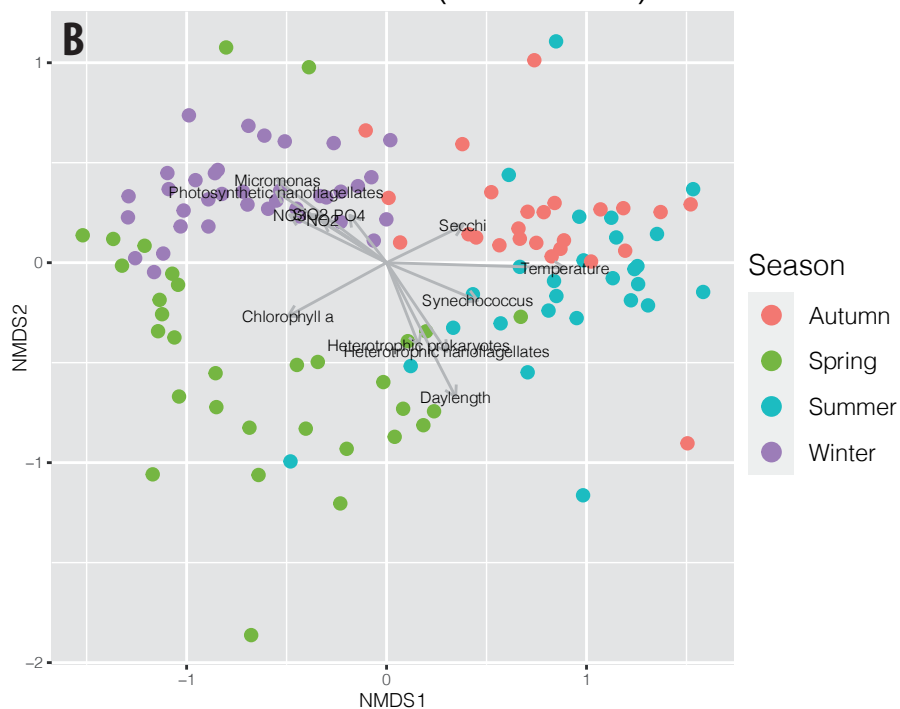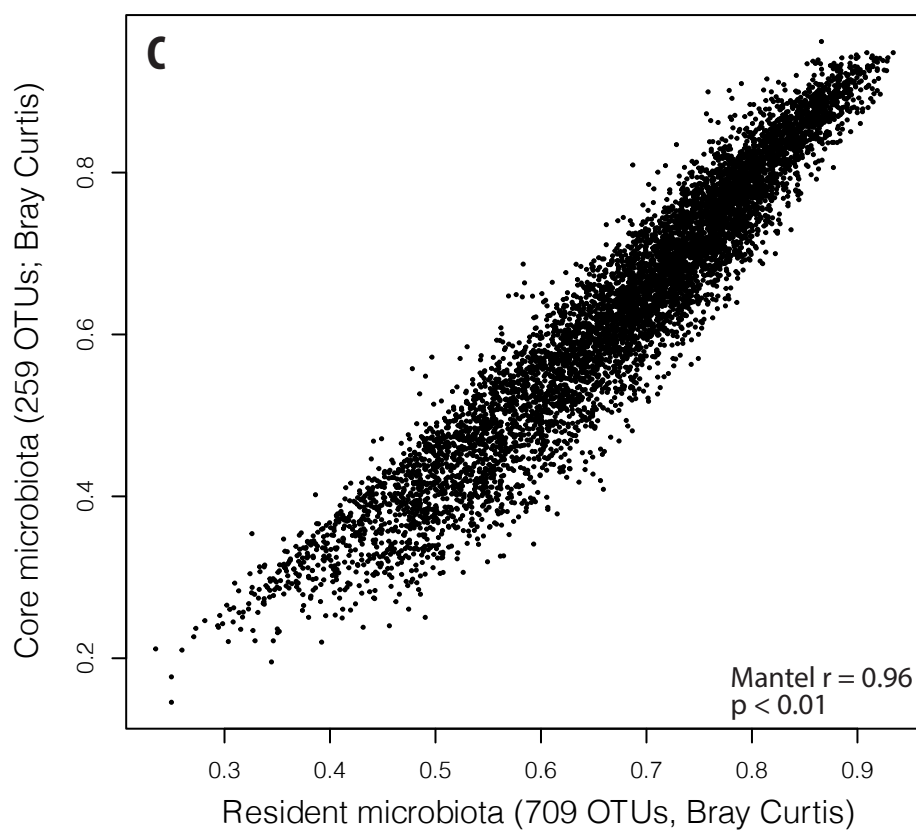

Supplement: Supplementary file 11 — Additional file 11: Figure S10. Panels A and B: NMDS based on Bray Curtis dissimilarities of communities including resident (Panel A) and core (Panel B) OTUs, to which environmental variables were fitted. Only variables with a significant fit are shown (p < 0.05). Arrows indicate the direction of the gradient, and their length represents the strength of the correlation between OTUs and an environmental variable. The color of the samples (circles) indicates the season to which they belong. Panel C: Relationship between Bray Curtis distances of the resident and core microbiotas. Results of the Mantel test (coefficient and significance indicated in the figure) indicate that both distance matrices are highly and significantly correlated. [file 40793_2022_417_MOESM11_ESM.pdf]

## Resident OTUs

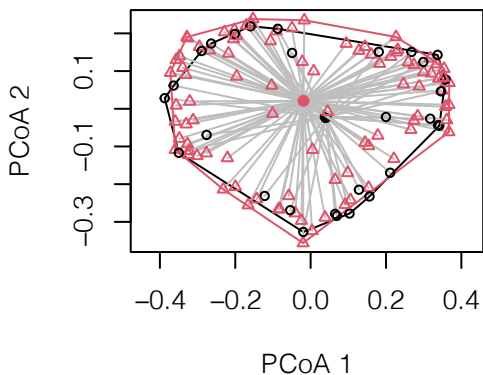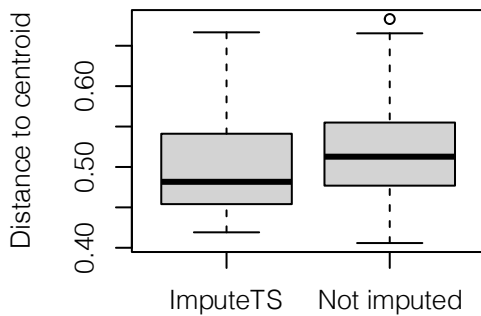

## Core OTUs

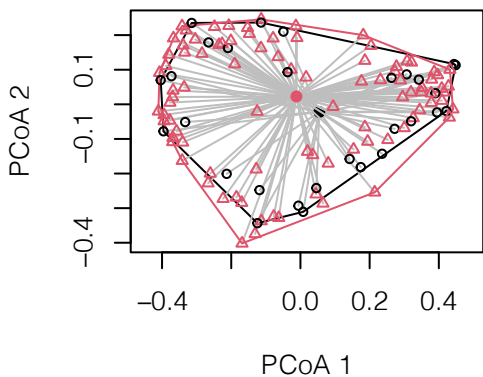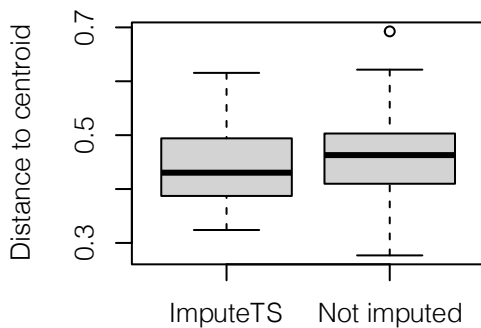

Supplement: Supplementary file 12 — Additional file 12: Figure S11. Betadispersion analyses based on Bray–Curtis dissimilarities for resident (upper panel) and core (lower panel) OTUs originating from both pico- and nanoplankton samples, with or without imputed values as implemented in imputeTS. These two groups (imputed vs. non-imputed) did not display significant differences (permutest p > 0.05) in their betadispersion. The red triangles indicate samples without imputed values, while black circles indicate those including imputed values. Samples including resident or core OTUs with or without imputed values are also shown in the boxplots on the right. [file 40793_2022_417_MOESM12_ESM.pdf]

# Resident OTU abundance vs. occurrence

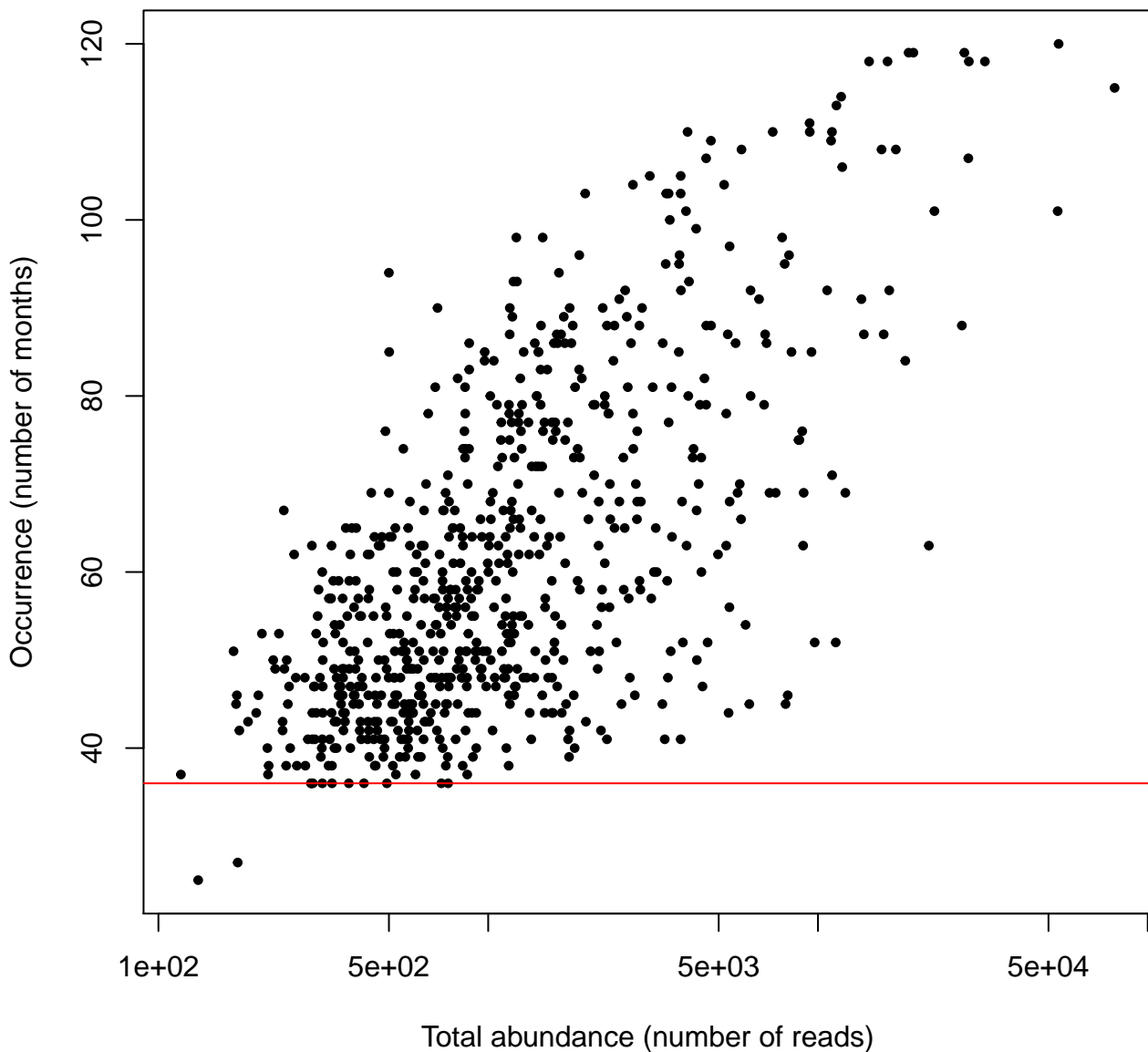

Supplement: Supplementary file 13 — Additional file 13: Figure S12. Occurrence of resident OTUs in number of months as a function of their total abundance in number of reads. The red line indicates the 36-month occurrence threshold for resident OTUs. Only two OTUs (bn_000846 and bn_000692) out of 709 present in 25 and 27 months respectively were kept in the resident microbiota given that their presence or absence appeared to be influenced by the missing value imputation approach as implemented in imputeTS. [file 40793_2022_417_MOESM13_ESM.pdf]

## Eigenvalues of significant dbRDA axes

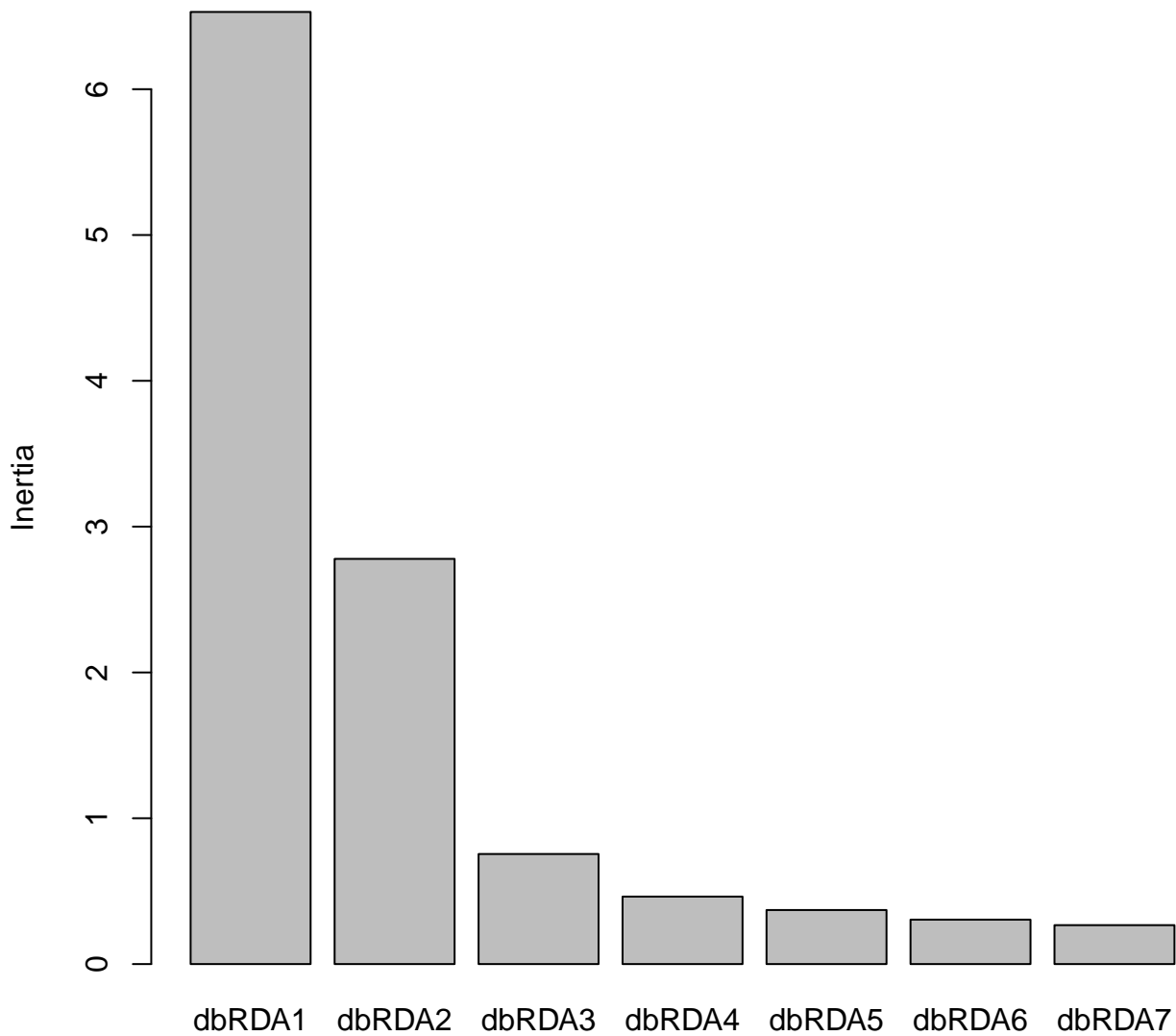

Supplement: Supplementary file 14 — Additional file 14: Figure S13. Significant dbRDA axes (p < 0.01) and the amount of variance explained by each. Note that the first two dbRDA axes explain ca. 80% of the variance. [file 40793_2022_417_MOESM14_ESM.pdf]
