# Supplementary material for: Long-term patterns of an interconnected core marine microbiota
Source: Environ Microbiome. 2022 May 7;17:22. doi: 10.1186/s40793-022-00417-1 (PMC9080219; doi:10.1186/s40793-022-00417-1)
Supplement: Supplementary file 7 — Additional file 7: Figure S6. Distribution of the Pearson Correlation Coefficient in the core network. [file 40793_2022_417_MOESM7_ESM.pdf]

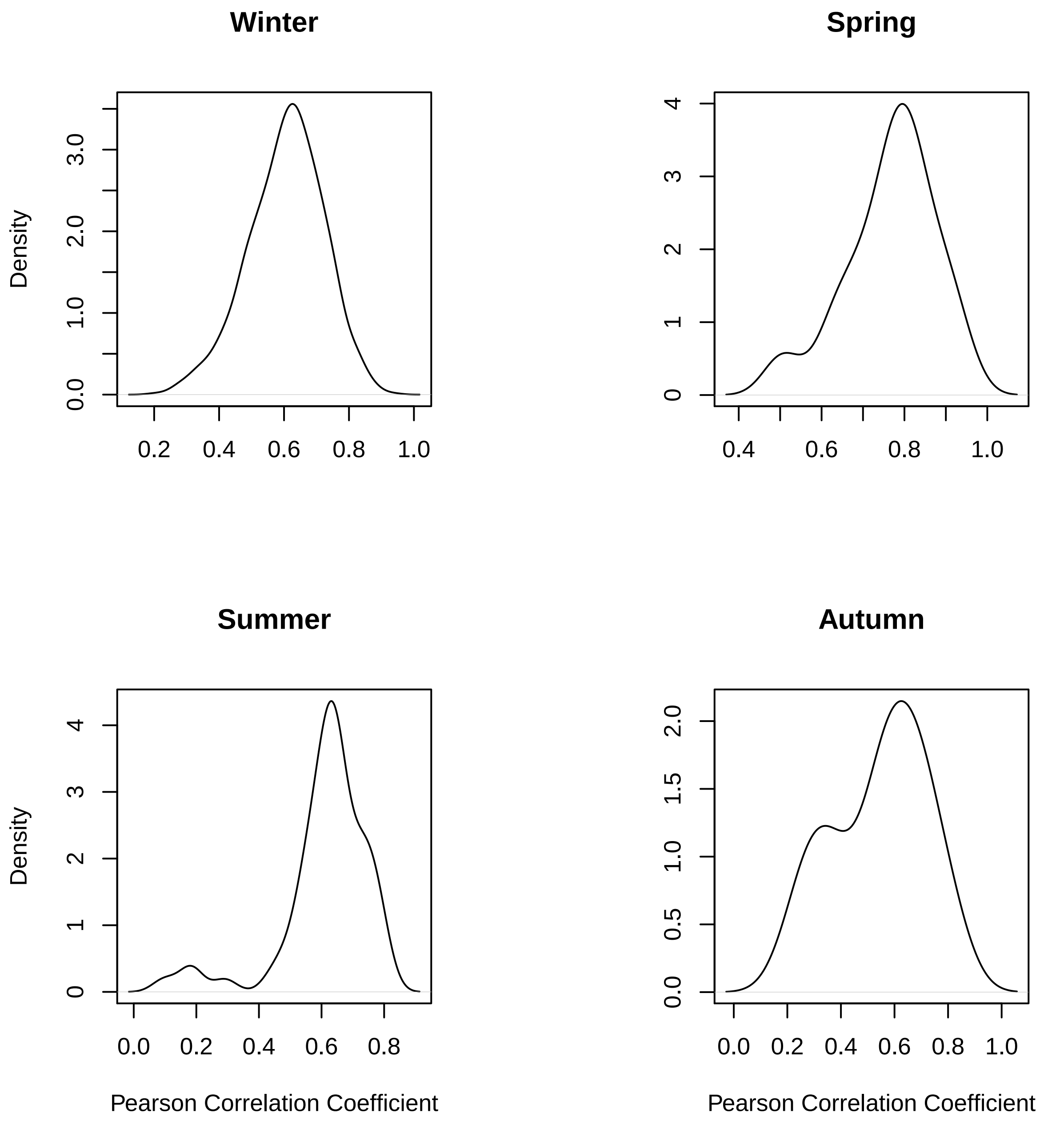

Winter
Spring
Summer
Autumn
Density
Density
Pearson Correlation Coefficient
Pearson Correlation Coefficient
